# Zinc Hyperaccumulation in Squirrelfish (*Holocentrus adscenscionis*) and Its Role in Embryo Viability

**DOI:** 10.1371/journal.pone.0046127

**Published:** 2012-10-04

**Authors:** E. David Thompson, Gregory D. Mayer, Chris N. Glover, Tom Capo, Patrick J. Walsh, Christer Hogstrand

**Affiliations:** 1 Department of Biological Sciences, Northern Kentucky University, Highland Heights, Kentucky, United States of America; 2 The Institute of Environmental and Human Health, Department of Environmental Toxicology, Texas Tech University, Lubbock, Texas, United States of America; 3 School of Biological Sciences, University of Canterbury, Christchurch, New Zealand; 4 Division of Marine Biology and Fisheries, Rosenstiel School of Marine and Atmospheric Sciences, University of Miami, Miami, Florida, United States of America; 5 Department of Biology, University of Ottawa, Ottawa, Ontario, Canada; 6 Diabetes and Nutritional Sciences Division, King's College London, London, United Kingdom; Glasgow Caledonian University, United Kingdom

## Abstract

Female squirrelfish (Fam. Holocentridae) can accumulate and temporarily sequester copious amounts of zinc (Zn) in their livers. There, it is initially compartmentalized before a subsequent, estrogen-triggered redistribution to the ovaries. Here we show that cellular uptake of Zn is also influenced by estrogen signaling, and that estrogen increases concentrations of the plasma Zn-binding protein vitellogenin (VTG). However, estrogen-mediated increases in VTG are not sufficient to accommodate the magnitude of hepato-ovarian Zn transfer in female squirrelfish (*Holocentrus adscensionis*). These findings suggest that holocentrids have acquired the ability to use hormonal cues to drive hepatic uptake and storage of Zn, signal for its physiological redistribution, and influence the capacity for systemic transport of Zn beyond the mediation of increased plasma VTG concentrations. Such specific adaptations suggest an advantage for the oocyte, which is corroborated in further studies where we determined that oocyte Zn concentrations are positively correlated with egg viability in captive-spawned squirrelfish. The novel nature of these findings underlies the importance of Zn in squirrelfish reproductive biology.

## Introduction

Zinc (Zn), the second most abundant micronutrient in vertebrates after iron, is necessary for processes involving transcription, enzyme structure and activity, antioxidant defense, maintenance of membrane integrity, and cell signaling [1]. However, at elevated levels Zn can become toxic, particularly in aquatic organisms. To counteract the potential toxicity of Zn, organisms usually maintain defined concentrations of biologically active intracellular Zn required for normal function. This is achieved via regulation of membrane-bound Zn-transporters, such as those of the ZIP and ZnT families of integral membrane proteins (Slc39; Slc30), and intracellular proteins that chelate Zn such as the metal-binding protein metallothionein (MT) [2], [3].

The squirrelfish family, Holocentridae, presents an interesting and useful system for studies of Zn biology. Members of this coral reef fish family contravene typical patterns for Zn regulation and accumulation. Remarkably, females of Fam. Holocentridae can accumulate concentrations of Zn in liver tissue up to 500 times higher than what is typical for vertebrates [4], [5]. In contrast, male squirrelfish do not display elevated Zn levels, and hyperaccumulation is restricted to the liver and ovaries of the female. Increased hepatic MT concentrations always accompany elevated hepatic Zn levels, where MT has been shown to bind up to 70% of all Zn present in the liver depending on Zn load [4]. In individual females with high hepatic Zn levels, MT was estimated to constitute 35% (w/w) of all hepatic proteins [5], [6]. Previous findings suggest that the MT protein sequence deduced from squirrelfish MT cDNA is very similar to that of other vertebrates [7]. Thus, it is unlikely that Zn accumulation in female squirrelfish liver is caused by an atypical MT structure.

Studies of the reproductive biology of squirrelfish indicate that spawning is synchronous, with mature oocytes released more than twice during the spawning season, and as such has been characterized as batch spawning [8]. Increased concentrations of Zn in the liver of female squirrelfish at the Florida Keys are known to occur primarily from August through April, while Zn levels are indistinct to those of males during May through July [5], [9]. In a study of the yearly reproductive cycle of female squirrelfish, liver Zn concentration peaked during two separate times of the year (Aug, Dec) [9]. Ovarian Zn levels peaked at two separate instances (Nov, Mar) and these increases in ovarian Zn seemed to overlap with the return of liver Zn to normal values, indicating a shift of Zn from the liver to the ovary. In addition, plasma Zn levels increased during the timeframe when liver Zn was beginning to decline, and while ovarian Zn was beginning to accumulate, indicating a systemic shuttling mechanism from the liver to the ovaries. It is well-known that the hepatically-derived yolk precursor protein vitellogenin (VTG) is a Zn-binding protein [10]. Thus VTG is a potential vehicle for Zn transport between liver and ovaries of female squirrelfish and, as expected, plasma VTG concentrations are elevated during the egg-producing periods of the female squirrelfish reproductive cycle [9]. Furthermore it has also been shown that female squirrelfish have a higher rate of intestinal Zn absorption than males, and this additional Zn is initially accumulated in the liver [11].

All available evidence suggests that the accumulation of Zn in these animals provides a biological advantage, and its redistribution from the liver to the ovaries is related to reproduction. However there are still large gaps in our knowledge of why and how squirrelfish accumulate Zn. The purpose of this study, therefore, is to further explore the unique Zn homeostasis of the female squirrelfish reproductive cycle by examining: (1) Zn uptake into the liver, (2) the transport of Zn from the liver to the ovaries, (3) the destination of the accumulated Zn once it is delivered to the ovaries, and (4) the importance of Zn for successful development of the embryos. The massive hepatic Zn accumulation observed in Holocentridae appears to be achieved, at least in part, by a high capacity of females to transport Zn into hepatocytes [5]. Previous studies indicate that this difference between sexes is not the result of separate diets or any other exogenous factor but rather a response to endogenous factors related to reproduction, suggesting this phenomenon may be hormonally regulated [5], [7], [9]. To test the hypothesis that Zn transport into female squirrelfish hepatocytes is mediated by female-specific reproductive hormones, we chose to use primary cultures of squirrelfish hepatocytes to explore the potential interplay between Zn and the female reproductive hormone, 17 β-estradiol (E_2_), in relation to the hepatic uptake of Zn. Previous studies of the plasma molar concentrations of both Zn and VTG in female squirrelfish indicate that circulating squirrelfish VTG would need to bind as many as 11 Zn atoms per dimer [9], [12]. To test the hypothesis that squirrelfish VTG is unique in this regard, we isolated and characterized squirrelfish VTG, in terms of size and Zn content, to determine if it was exceptional in its ability to bind and transport Zn in comparison to other studied VTG's. While the exact purpose of this unusual Zn physiology for squirrelfish has remained enigmatic, our hypothesis has been that female squirrelfish ultimately accumulate this Zn first in the liver, and then the ovaries to make it available for the proper development of offspring. To test this, we examined spawns from squirrelfish bred in captivity in order to determine if the ovarian Zn accumulated by female squirrelfish is passed on to the oocyte, and to assess the importance of Zn content on embryo viability. Thus the aims of the present study were to find mechanisms by which female squirrelfish can specifically accumulate Zn in the liver and transport it to the ovaries, and to investigate if squirrelfish accumulate Zn to improve spawn viability.

## Results

### Squirrelfish Hepatocyte Zinc Flux

**Figure 1:**
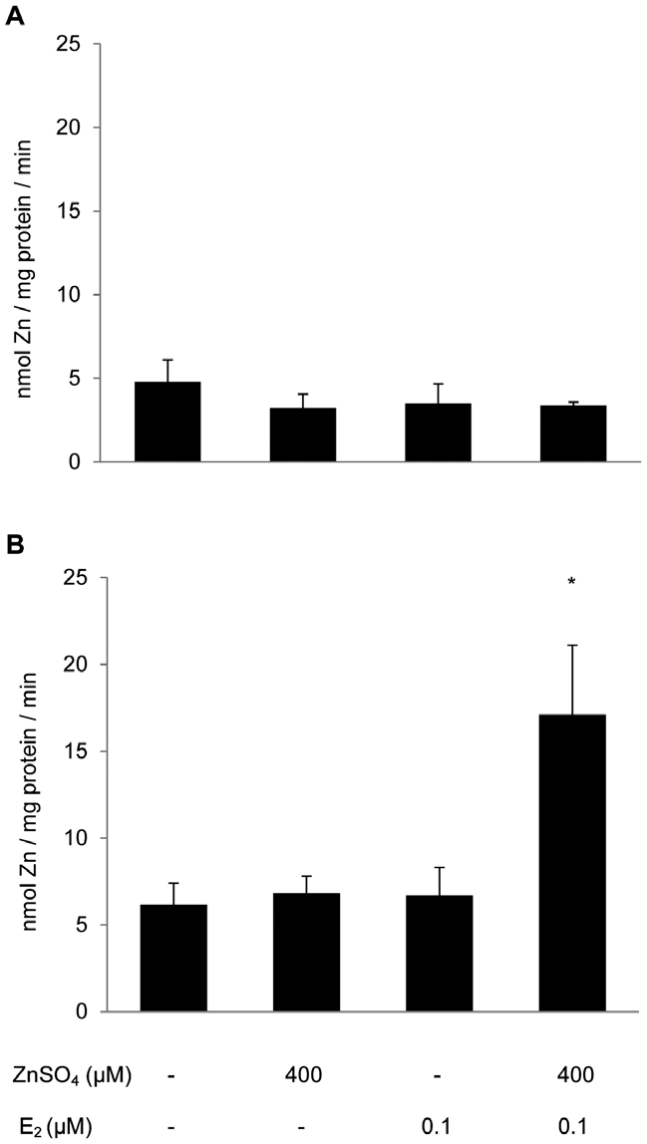
Zinc influx in squirrelfish hepatocytes. Comparison of zinc influx in (A) male and (B) female squirrelfish control hepatocytes, and those pre-treated with 400 µM Zn, 0.1 µM E_2_, and 400 µM Zn+0.1 µM E_2_, then washed twice, and subjected to Zn influx analysis in presence of 150 µM Zn and ^65^Zn as tracer. Values are expressed as arithmetic means with error bars indicating SEM (n = 2–3 for male squirrelfish and 3–5 for female squirrelfish). Post-hoc comparisons when warranted by ANOVA were conducted via Tukey's HSD test. * denotes values that are significantly different than controls (p<0.05).

Zn influx at 150 µM was quantified in male squirrelfish hepatocytes pre-treated with 400 µM Zn, 0.1 µM E_2_, or 400 µM Zn+0.1 µM E_2_, as compared to control (untreated) male hepatocytes (Figure 1A). The data were not significantly different (ANOVA; p = 0.744). Zn uptake in control male squirrelfish hepatocytes was found to be 4.79±1.32 nmol Zn/mg protein/min. Zn uptake in males pre-treated with 400 µM Zn alone was 3.21±0.85 nmol/mg/min. Males pre-treated with 0.1 µM E_2_ exhibited Zn influx of 3.50±1.17 nmol/mg/min. Male squirrelfish that were pre-treated with both 400 µM Zn and 0.1 µM E_2_ exhibited Zn influx of 3.37±0.21 nmol/mg/min.

Additionally, Zn uptake at 150 µM was quantified in female squirrelfish hepatocytes subjected to pre-treatments with 400 µM Zn, 0.1 µM E_2_, or 400 µM Zn+0.1 µM E_2_, as compared to control (untreated) female hepatocytes (Figure 1B). The data were significantly different (ANOVA; p = 0.047). Zn uptake in control female squirrelfish hepatocytes was 6.16±1.23 nmol/mg/min. In females treated with 400 µM Zn alone, the observed Zn influx of 6.84±0.96 nmol/mg/min was not different from control females (p = 0.992). Females pre-treated with 0.1 µM E_2_ exhibited Zn influx (6.69±1.61 nmol/mg/min) that was not different from controls (p = 0.995). Notably, female squirrelfish that were pre-treated with both 400 µM Zn and 0.1 µM E_2_ exhibited a significant two-fold increase in Zn influx (14.1±3.68 nmol/mg/min) when compared to control females (p = 0.048). It should be noted that Zn influx in control female squirrelfish hepatocytes was not found to be different from that in male controls (p = 0.501).

### Squirrelfish VTG Isolation and Characterization

**Figure 2:**
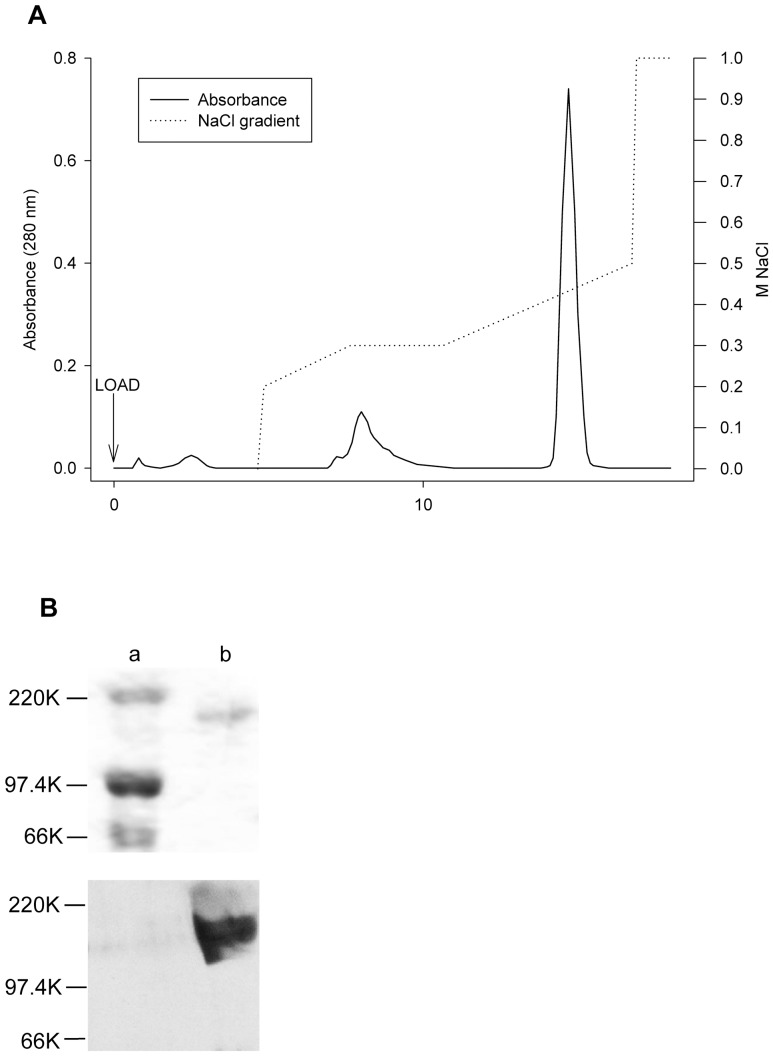
Purification and characterization of squirrelfish VTG. (A) Purification of VTG from plasma of E_2_-injected squirrelfish subjected to anion-exchange chromatography; and (B) a typical dehydrated Coomassie-stained polyacrylamide gel and western blot for squirrelfish VTG. The stepwise NaCl gradient introduced to the column to isolate VTG was as follows: 0–25 mL, 0.00 M NaCl; 26 mL, 0.20 M NaCl; 40–55 mL, 0.30 M NaCl; 85 mL, 0.50 M NaCl; 86 mL, 1.00 M NaCl. Elution samples were collected in 5-mL fractions following NaCl gradient introduction. Absorbance was measured at 280 nm. The contents of the gel and western blot lanes are as follows: (a) molecular weight marker, (b) squirrelfish plasma sample. The molecular weight marker as shown consisted of: myosin-220K, phosphorylase B-97.4K, bovine serum albumin-66K.

Figure 2A represents an elution profile of E_2_-treated squirrelfish plasma subjected to anion-exchange chromatography. Following removal of unbound proteins, two primary protein peaks were eluted from the Hi-Trap Q column, as evidenced by 280 nm absorbance readings taken while fractions were collected. The first peak eluted at around 0.30 M NaCl in control and E_2_-treated squirrelfish. The second peak eluted from the column at 0.41 M NaCl. This second peak was absent in samples from control squirrelfish, therefore it can be assumed that this plasma protein fraction exists as a result of the influence of E_2_.

E_2_-specific fractions obtained by anion-exchange chromatography were subjected to SDS-PAGE and subsequent Coomassie staining to determine the molecular weight of each protein (Figure 2B). The protein samples obtained from these fractions had a molecular weight of 197.3±2.2 kDal. Furthermore, only one protein band was observed for each fraction, verifying that the protein contained within the fraction was homogeneous. Figure 2B also shows a representative western blot for squirrelfish VTG from E_2_-treated squirrelfish plasma. Based on the outcome of the western analysis and the single band observed in the Coomassie-stained gels, the E_2_-specific fractions obtained from squirrelfish plasma samples were identified as VTG. The purified VTG samples were then subjected to atomic absorption spectroscopy analysis to determine the molar concentrations of any Zn that may be bound to each protein subunit. Squirrelfish VTG was shown to be a Zn-containing protein, with a molar ratio of 1.12±0.17 Zn atoms per VTG subunit.

### Squirrelfish Embryo Viability and Zinc Content

**Figure 3:**
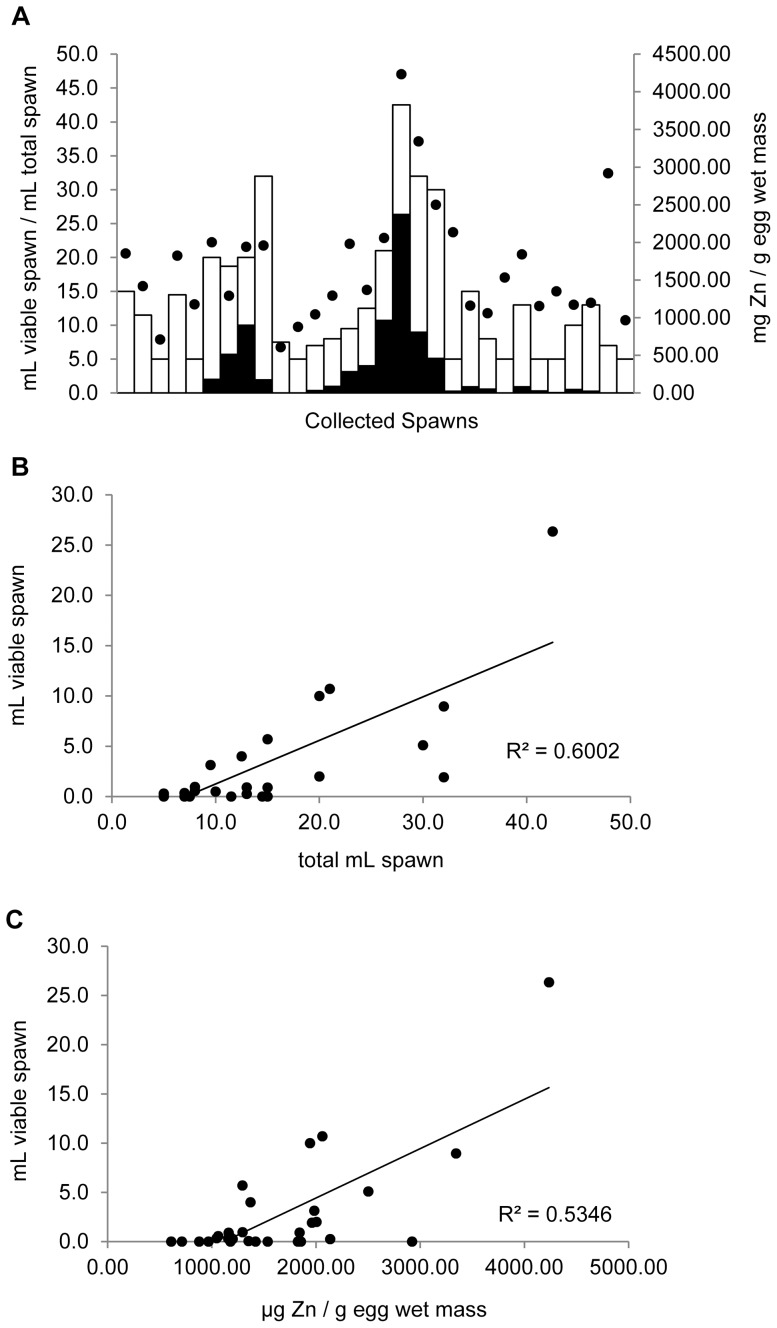
Zinc content in squirrelfish eggs and embryo viability. (A) Collections of individual spawns taken from squirrelfish bred in captivity, sorted by date and measured for total spawn volume (entire bars), viable spawn volume (filled portion of bars), and egg Zn content; and the relationship of egg viability to (B) total spawn volume and (C) egg Zn content. Significance of linear regressions was determined by ANOVA (p<0.05).

Squirrelfish were allowed to spawn in captivity, and each spawn was assessed for volume, egg viability, and egg Zn content (Figure 3A). The collected spawn volumes ranged from 5.0 mL to 42.5 mL, with an average of 13.5±1.77 mL. Squirrelfish embryo viability was significantly related to spawn volume (Fig. 3B; r^2^ = 0.600; p<0.001). Embryo viability was also significantly related to Zn content (Fig. 3C; r^2^ = 0.535; p<0.001). The average Zn content of squirrelfish eggs was found to be 1668±144 µg Zn/g egg, and the minimum concentration of Zn that provided for any viable eggs was 1000 µg Zn/g egg. It should be noted that spawn volume was also significantly correlated to Zn content (r^2^ = 0.573; p<0.001).

## Discussion

Cellular Zn influx and efflux are regulated by changing expression and activities of Zn transporters [3]. There are two protein families dedicated to Zn movement across lipid bilayers in animals, the ZIP and ZnT families of integral membrane proteins (Slc39; Slc30). At least 13 Zn transporters have been found to be expressed in the model teleost zebrafish (*Danio rerio*) [13]. Many Zn transporters are regulated by cellular Zn status, but there is also endocrine control over Zn levels. For example, in fish the calcium regulatory hormones stanniocalcin and calcitriol decrease and increase Zn uptake, respectively [14], [15]. Also El-Tanani and Green [16] presented evidence from a study in the human MCF-7 breast cancer cell line in which E_2_ administration increased expression of ZIP6. Previous findings in squirrelfish suggest that E_2_ alone is not the mediator of the initial accumulation of hepatic Zn [5], [7], [12]. In the present study a significant increase in the influx of Zn was observed in female squirrelfish hepatocytes during exposures to a combination of E_2_ and Zn, suggesting a co-operative effect of Zn and E_2_ on the uptake of Zn. These results suggest the possibility of a regulated Zn transport mechanism associated with the livers of female squirrelfish that allows for sex-specific hepatic Zn accumulation to occur during the female squirrelfish reproductive cycle. The exact nature of the transporters associated with cellular Zn uptake in the squirrelfish remains unstudied. It will be interesting to examine if the combination of Zn and E_2_ has the ability to affect sex-specific differential expression of Zn transporters in tissues of female squirrelfish *in vivo*.

Squirrelfish VTG was found to contain one Zn atom per monomer. Previous reports in both red drum (*Sciaenops ocellatus*) and *Xenopus* have established VTG as a Zn protein in these species, with ratios of 3 and 1 Zn atom(s) per VTG monomer, respectively (Table 1). Thus, the present study indicates that squirrelfish VTG is not unique, in that 2 Zn atoms are typically bound in the native state of the dimer. It was considered that weakly-bound Zn might have dissociated from VTG during isolation of the protein. However, such loosely associated Zn would not be biologically relevant as the bonds would not be stable in the plasma and it would therefore not allow VTG to serve as a specific vehicle for Zn transport to the ovaries. These findings suggest that one or more additional proteins, or other ligands, are required to account for hepato-ovarian transport of the additional plasma Zn observed from previous studies of the molar concentrations of VTG and Zn in E_2_-treated squirrelfish.

**Table 1:** Comparison of squirrelfish VTG from the present study (mean ± SEM) with the VTG's of other studied species in terms of size and Zn content.

Species	Mol. Wt. (KDal)	Zn (atoms/VTG monomer)
*Holocentrus adscensionis*	197±2.21	1.1±0.2
*Oncorhynchus mykiss* a	170	-
*Gadus morhua* a	167	-
*Sciaenops ocellatus* b	225	3
*Xenopus laevis* c	220	1

a [39].

b [40].

c [41].

Other Zn-binding proteins have been identified within plasma, one of the most abundant being albumin. However albumin is not preferentially incorporated by the developing oocyte [17]. Although albumin could act as a Zn shuttle, donating Zn to follicular transporters, E_2_ is known to exert negative effects on the circulating levels of serum albumin by reducing transcription of the albumin gene and also by reducing the half-life of albumin mRNA [18]. The positive or neutral effects of E_2_ discerned in the present study therefore suggest that albumin is unlikely to be a significant mediator of liver-to-ovary Zn transport. Novel plasma Zn-binding proteins have been indicated in both albacore tuna (*Thunnus alalunga*) and channel catfish (*Ictalurus punctatus*) [19], [20]. In winter flounder (*Pseudopleuronectes americanus*), yolk proteins exist within the oocyte that are derived from precursors distinct from VTG [21]. Perhaps such a plasma Zn-binding protein exists in squirrelfish that is incorporated by the developing oocyte, and also supplies Zn in addition to that contributed by VTG. There is also the possibility of Zn transport to the developing oocyte being facilitated by the presence of low molecular weight ligands in the maternal plasma. For example, amino acids such as histidine and cysteine have high binding affinities for Zn, and are known to enhance the maternofetal transfer of this element in mammals [22]. Additionally, histidine and cysteine have both been shown to influence intestinal Zn absorption in fish systems [23]. However, it is not known whether these, or other, potential Zn-binding ligands increase during the reproductive cycle in squirrelfish.

Data presented in this study illustrate that squirrelfish embryo viability is dependent upon Zn content within the egg. This may not by itself be remarkable, but the fact that a Zn content of 1 to 4 mg/g wet mass was present in viable squirrelfish spawns is quite astonishing considering that this is 50 to 400 times the Zn level found in oocytes from other fish species (Table 2). Zn is required for meiotic completion during oocyte development in mice, and Zn content is known to increase during oocyte development in zebrafish by as much as 350% [24], [25]. Furthermore it is known that Zn is necessary for embryonic development. For example in a microarray experiment using zebrafish ZF4 cells and siRNA specific to metal-regulatory transcription factor 1 (MTF-1), Hogstrand *et al.* [26] identified over 20 potential MTF-1 target genes that code for transcription factors and other proteins involved in embryonic development, and which could therefore be induced by the presence of Zn. Another study in zebrafish presented evidence that the longitudinal migration of stem cells and extension of the body axis is dependent on the presence of a particular Zn importer, Zip6, and silencing of Zip6 resulted in a dwarfed embryo [27]. Furthermore the growth-promoting effect of Zn in embryonic fish has been illustrated by retardation of growth during Zn deficiency [28], [29].

**Table 2:** Comparison of Zn concentrations in squirrelfish oocytes (viable and non-viable) from the present study with the average Zn content of oocytes from other studied species.

Species	Zn (µg/g wet mass (mean))
*Holocentrus adscensionis*	430–4230 (1660)
*Catostomus commersoni* a	17–27†
*Salmo salar* b	19–35
*Cyclopterus lumpus* c	9.9–12.9 (11.8)
*Gadus morhua* c	17.6–27.0 (22.0)
*Alburnus alburnus* d	(25)
*Chelon labrosus* e	(10)
*Mallotus villosus* f	14.5–16.5 (15.5)

Data are shown as range and/or mean within parentheses.

a [42].

b [43].

c Danish Food Composition Database.

d Swedish Food Database.

e Banca Dati Di Composizione degli Aliment per Studi Epideminologici in Italia.

f The Icelandic Food Composition Database.

† Data reported as mg/kg dry weight. Dry weight/wet weight ratio of 4 applied.

The average Zn concentration in squirrelfish eggs was found to be 1668±144 µg/g egg, and as such the Zn content of the squirrelfish egg seems to be in line with the massive amounts of Zn accumulated in the female squirrelfish liver and ovary. It has been suggested that female squirrelfish supply large amounts of Zn to larvae for proper eye development and function. Zn is highly abundant in most parts of the eye and is found in particularly high concentrations in the tapetum lucidum, the layer of the eye present in many nocturnal organisms that improves vision in low light conditions [30]. Zn may also contribute to the maintenance of glutamate stores under prolonged periods of darkness [31]. Another possibility is that the addition of large amounts of Zn to the eggs enhances the chances of a successful hatch. In this study elevated levels of Zn (≥1000 µg/g) were necessary for viability of the squirrelfish egg. This effect could occur through the use of Zn in high amounts during larval development, or via any antimicrobial properties of Zn [32]. It should also be considered that the high Zn levels deposited in squirrelfish eggs may serve as a deterrent to potential predators. Metals are known to cause avoidance behaviors in fish, and at least in the case of waterborne Zn, if given the choice between clean and Zn-supplemented water in an experimental system, fish prefer the clean water [33]. Whether any of these factors, and/or others, necessitate the ability for female squirrelfish to accumulate such large amounts of Zn remains to be ascertained.

Hopefully, by studying natural adaptations that allow for unique accumulation and transport of trace minerals such as Zn, science can be better informed as to the mechanisms governing Zn hyperaccumulation in disease. In recent years, dysregulation of Zn has been found to be a major contributor to several prominent human diseases and conditions, such as cancer, diabetes, age-related macular degeneration, immunodeficiency, Alzheimer's disease, and neuronal death during post-perfusional ischemia. Studies of how squirrelfish manage to use Zn as a macronutrient as opposed to a trace element might help us to understand Zn dysregulation in these diseases. However, for this to be realised, the molecular networks that are involved in Zn regulation in squirrelfish need to be uncovered.

## Materials and Methods

### Ethics Statement

All fish used in this study were manipulated according to the National Institute of Health Guide for Care and Use of Laboratory Animals, and prior approval of experimentation was granted by the University of Miami Institutional Animal Care and Use Committee (protocol # 03-185; assurance # A-3224-01).

### Animal Husbandry and Experimentation

Squirrelfish (*Holocentrus adscensionis* Osbeck 1765) were wild-captured in hand-held nets by SCUBA divers on reefs near Tavernier, Florida, USA. Male and female squirrelfish (150–300 g) were transported to the Rosenstiel School of Marine and Atmospheric Sciences (RSMAS), University of Miami, where they were housed in tanks (4000 L) supplied with a continuous flow of aerated seawater (28°C) from Biscayne Bay until experimentation. Fish were fed daily to satiation with live shrimp and were allowed to acclimate to laboratory conditions. To ameliorate suffering, fish were anesthetized with MS222 prior to experimentation. For all aspects of this study, male and female squirrelfish were distinguished by gonadal examination. Unless otherwise noted all chemicals were obtained from Sigma Aldrich, Inc.

Squirrelfish to be used specifically for breeding purposes were captured and transported to RSMAS as described previously, where they were acclimated at the experimental hatchery for one month before being transferred to 80,000 L tanks containing artificial reef structures (constructed from natural materials also collected near Tavernier, FL) in flow-through seawater. Twenty-five squirrelfish were moved to the artificial reef structure tank where daylight/dark and moon phase cycles were applied to optimize breeding conditions.

### Hepatocyte Zn Fluxes

Zn fluxes were assessed in primary squirrelfish hepatocytes as described by Hogstrand *et al.* [5] with modifications. Briefly, squirrelfish were anesthetized in MS222 (0.5 g/L) until loss of dorso-ventral equilibrium became apparent. Fish then underwent surgery where cannulation of the bulbus arteriosis allowed retrograde perfusion of the liver with modified Hank's buffer (136 mM NaCl, 5.4 mM KCl, 0.81 mM MgSO_4_, 0.44 mM KH_2_PO_4_, 0.33 mM Na_2_HPO_4_, 5.0 mM NaHCO_3_, 10 mM 4-(2-hydroxyethyl)-1-piperazineethanesulfonic acid (HEPES), and 3 mM glucose adjusted with NaOH to a pH of 7.6 at room temperature) that included freshly prepared collagenase (0.4 mg/mL). Hepatocytes were isolated from male and female squirrelfish by massaging perfused liver tissue through a sterile, 100 µM mesh and then seeded at a density of 4×10^6^ cells/mL in 25 cm^2^ flasks (Falcon T25 Primeria) with 7 mL of Leibovitz's L-15 medium supplemented with fetal bovine serum (FBS; 10%; Gibco), penicillin and streptomycin (50 µg/mL each) and fungizone (2.5 µg/mL).

On day 2 post plating, cells were treated with 0.01–1 µM 17ß-estradiol (E_2_; dissolved in ethanol for a final EtOH concentration of 1∶1000 in media) and/or 200–400 µM of ZnSO_4_ in renewed culture medium for 48 hours. Preliminary experiments showed that there were no qualitative differences in results obtained from treatment of cells within these concentration ranges of E_2_ and ZnSO_4_, and subsequent experiments therefore included only single concentrations of E_2_ (0.1 µM) and/or ZnSO_4_ (400 µM). After treatments with E_2_ and/or Zn, hepatocytes were washed twice with 5 mL modified Hank's medium, as above, without the addition of collagenase. Cellular influx of Zn was studied by incubation of hepatocytes for 10 min in 2 mL of a ‘flux medium’ containing 150 µM ZnSO_4_, 170 mM NaCl, 1 mM CaCl_2_, 0.81 mM MgCl_2_, 10 mM HEPES, pH 7.5, with ^65^Zn added as a radiotracer (∼0.1 µC_i_/mL; n = 2–5 fish). ^65^Zn was quantified as counts per minute (CPM) by gamma counting (Packard Cobra II). Zn fluxes were stopped by two washes with 5 mL ‘flux medium’ without ^65^Zn and with 10 mM ethylenediaminetetraacetic acid (EDTA) added as a chelating agent to remove adsorbed ^65^Zn. Finally the cells were solubilized in 1 mL of 1 M NaOH. Following the addition of 200 µL of 6 M HCl, samples were analyzed for protein content using the Bradford assay with bovine serum albumin standards [34]. Specific activity of ^65^Zn was calculated, and the amount of Zn accumulated was quantified and normalized to total soluble protein content (nmol Zn/mg protein/min).

### Isolation of VTG

To induce VTG synthesis, squirrelfish were given three intraperitoneal injections of 5 mg E_2_/kg body weight (1 mL/kg in peanut oil). Following E_2_ injections squirrelfish were subjected to euthanasia by treatment with MS222. Approximately 2 mL of blood was withdrawn from the caudal vessels with a heparinized syringe and plasma was separated from blood cells by centrifugation (14000×*g*) for 3 min. The plasma was divided into 0.5 mL aliquots, flash frozen in liquid nitrogen, and immediately stored at −80°C. Plasma samples from E_2_-treated squirrelfish were subjected to SDS-PAGE via the protocol of Laemmli [35]. The protein concentration for each sample was determined by Bradford assay and samples were diluted with distilled water as necessary. Samples were diluted 1∶4 with sample buffer [62 mM Tris-HCl, pH 6.8, 10% glycerol, 5.0% 2-mercaptoethanol (added just before dilution), 2.0% SDS, 0.0012% bromophenol blue] and heated at 100°C for 5 min. A total of 25 µg of protein was loaded into each well. Electrophoresis was carried out on a 4% stacking gel and a 7.5% separating gel at 100 V for 2 h in a Mini-Protean II electrophoresis system (BioRad). Each gel also contained a molecular weight marker (Amersham Life Science). Following electrophoresis, proteins were transferred to nitrocellulose membranes for western analysis (Schleicher & Schuell) by electroblotting, as described by Towbin et al. [36], in a SemiPhor TE 70 semi-dry transfer unit (Hoefer Scientific). The blocking solution was 5.0% dehydrated nonfat milk in TBS-T (20 mM Tris-HCl, pH 7.4, 137 mM NaCl, 0.10% Tween-20). The primary antibody was a rabbit anti-turtle VTG IgG diluted 1∶10000 in TBS-T. The cross-reactivity of this antibody with squirrelfish VTG has been documented previously [7]. The secondary antibody was a horseradish peroxidase-linked donkey anti-sheep IgG (BioRad) diluted 1∶40000 in TBS-T. Typical series of washes with TBS-T were used following blocking and incubation with antibodies. Immunodetection was performed with an enhanced chemiluminescence system (ECL, Amersham) according to manufacturer's specifications. All incubations were carried out at room temperature.

E_2_-treated squirrelfish plasma samples containing VTG as confirmed by western blot were subjected to ion-exchange chromatography according to the protocol of Silversand and Haux [37], with minor modifications. Plasma samples (0.5 mL) were diluted 1∶10 in sample buffer A (20 mM Tris-HCl, pH 8.0). Following dilution, 0.5 mL of sample was injected onto the Hi-Trap Q 5-mL −N^+^(CH_3_)_3_ anion exchange column (Pharmacia Biotech). Before injection, the column was equilibrated with 25 mL of buffer A at 5 mL/min. Each sample that was injected onto the column contained 1% aprotinin (v/v) to minimize proteolytic degradation of plasma proteins. After injection, 25 mL of buffer A were run through the column (3×) to remove all unbound plasma components. Bound plasma proteins were separated from the column matrix via a stepwise NaCl gradient. The gradient was a mixture of buffer A with the elution buffer (buffer B – 1 M NaCl). The gradient was as follows: 0–25 mL, 100% A; 26 mL, 80% A, 20% B; 40–55 mL, 70% A, 30% B; 85 mL, 50% A, 50% B; 86 mL, 100% B. To remove all proteins that may have remained on the column, three column volumes (25 mL) of buffer B were run through the column. The flow-through rate through the column was 1 mL/min and 5 mL fraction volumes were collected. Each fraction was examined for protein content by measuring the absorbance at 280 nm as they were collected. The chromatographic procedure was carried out at 4°C to minimize further breakdown of plasma protein. Fractions were immediately frozen in liquid nitrogen and stored at −80°C.

### Determination of VTG Molecular Weight and Zn Content

Isolated fractions collected via anion-exchange chromatography were subjected to SDS-PAGE and western analysis as previously described. Following electrophoresis, protein bands were visualized after 15 min incubation in Coomassie Brilliant Blue protein stain (0.1% Coomassie Brilliant Blue, 50% methanol (v/v), 10% glacial acetic acid (v/v)). The molecular weight of each isolate was determined by comparisons with the molecular weight marker (n = 4). Additionally, purified fractions were subjected to acid digestion for determination of Zn content (n = 3). Samples were added to 2 mL of trace metal grade 70% HNO_3_ (Fisher Scientific), heated in a sand bath for 3 h at 120°C, and then cooled to room temperature before 0.5 mL of H_2_O_2_ was added. The temperature was then gradually increased to 120°C until all liquid had evaporated. The dried residues were reconstituted with 4 mL of 0.5% HNO_3_. These samples were then analyzed for Zn content by air/acetylene flame atomic absorption spectroscopy (Perkin Elmer, model 2380).

### Embryo Viability and Zn Determinations

Eggs from laboratory-bred squirrelfish were collected in a flow-through side port of the reef tank fed by rotational current of the flow-through seawater. A plankton net fitted inside the port was checked several times daily for eggs which were then washed from the collection apparatus and stored in clean seawater in graduated tubes. All spawns that were ≥5 mL were collected over a period of approximately six months. A total of 30 separate clutches were collected and analyzed as described. Percent egg viability was determined by placing eggs into a graduated cylinder with fresh seawater. Eggs that floated were considered viable, sinking eggs were not viable [38]. Zn analysis was conducted on digested egg samples via inductively coupled plasma optical emission spectroscopy (Varian Vista-MPX). Total egg mass from each collection was digested in 2 mL of trace metal grade HNO_3_ in a 50°C sand bath and residual nitric acid was allowed to evaporate. Each sample was then reconstituted in 5 mL of 0.50% HNO_3_ as stock and diluted accordingly before analysis to fall within range of the calibration standards.

### Data Analysis

Squirrelfish hepatocyte flux data were tested for normality via the Kolmogorov-Smirnov test (p = 0.087), and for equal variance via Levene's test (p = 0.066). Male and female control flux data were compared via Student's t-test. Trend analyses were conducted via ANOVA, and post-hoc comparisons, when warranted by ANOVA, were conducted via Tukey's HSD test. For the relationship of squirrelfish egg viability to Zn content, data were tested for normality via the Kolmogorov-Smirnov test (p = 0.060). Linear regressions were conducted, and significance was determined by ANOVA. Results of all statistical analyses were considered significant at p<0.05.
